# Dementia education in a primary school classroom: A pedagogical perspective

**DOI:** 10.1177/14713012221149760

**Published:** 2023-01-07

**Authors:** Louise Ritchie, Susan Henderson-Bone, Angela Gregory, Nick Jenkins

**Affiliations:** Alzheimer Scotland Centre for Policy and Practice, School of Health and Life Sciences, 6413 University of the West of Scotland, South Lanarkshire, UK; School of Education and Social Sciences, 6413 University of the West of Scotland, South Lanarkshire, UK; Alzheimer Scotland Centre for Policy and Practice, School of Health and Life Sciences, 6413 University of the West of Scotland, South Lanarkshire, UK; School of Education and Social Sciences, 6413 University of the West of Scotland, South Lanarkshire, UK

**Keywords:** children, dementia, education, pedagogy, primary school, video methods

## Abstract

The World Health Organisation recognises the importance of dementia education across all sectors of the population, including children. Previous research has shown that dementia education in schools has positively changed students’ knowledge about and attitudes to dementia, however little is known about the process of learning about a complex condition, such as dementia. This paper explores how pupils learn about dementia through a pre-planned educational session in the primary classroom to improve our understanding of the pedagogical approaches required for effective dementia education. Using a participatory video approach, 23 primary 6 pupils (aged 10–11) used iPads to film their experiences of a dementia education session using a resource called Understanding Dementia: Class in a Bag. These videos, along with researcher field notes were combined to create analytical vignettes to illustrate the learning processes and interactions that happened within the classroom. Two weeks later, the pupils were invited to a focus group to view footage of the session and reflect on their experiences and explore the understanding of dementia. The findings highlight the importance of embodied learning within the session, particularly in understanding the experience of living with dementia. By understanding the session from the pupil’s perspective, the findings show how pupils developed an empathetic understanding of dementia through play and felt more confident about engaging with and helping people living with dementia in future. This paper provides a new understanding of the process of learning about dementia for primary children, as well as demonstrating the viability of including dementia education within school curriculums internationally.

## Introduction

New approaches are required to improve understanding of dementia in the general population and reduce stigma (Swaffer, 2014; WHO, 2021). Dementia education has a role in increasing awareness and promoting an inclusive society for people with dementia, as well as increasing awareness of brain health and reducing the risk of developing dementia in later life (WHO, 2017).

It is estimated that 55.2 million people are living with dementia worldwide (WHO, 2021). Given the prevalence of dementia, it is inevitable that a significant proportion of children will, at some point, have a relative who is living with dementia. In addition, around 5% of those diagnosed with dementia will be under the age of 65 meaning that some children will have a parent who is diagnosed with dementia (Sikes & Hall, 2018).

Previous studies have highlighted the limited understanding of young people concerning dementia (Baker et al., 2018). Cowley (2005) found that 84% of British young people aged 11–14 years surveyed sourced their knowledge about dementia from the media, with some conflating the term dementia with the Harry Potter character––a ‘dementor’––or negatively identifying them as ‘weirdos.’ Yet other studies have indicated the desire of young people to improve their knowledge of dementia, particularly where individuals have a relative who is experiencing symptoms of the disease, where they cite feeling helpless or even blame (Celdrán et al., 2014).

Within the UK, there is no requirement for dementia education in the primary education curriculum. Where dementia is included, it is covered as a health and wellbeing topic and is usually the result of personal interest of the educators, collaboration with local universities or third sector organisations (Farina, 2020) or through intergenerational projects with local care homes (Di Bona et al., 2019). Dementia education in schools should aim to reduce stigma, increase understanding and promote helping behaviours. Central to this is giving children the opportunity to develop empathy for people with dementia, ensuring that they have insight into what a person with dementia may experience and the knowledge to respond appropriately (Baker et al., 2018).

Dementia education for children and adolescents has been evaluated positively, in terms of enjoyment (Farina et al., 2020b) and in improving children’s knowledge of, and attitudes towards, dementia (Baker et al., 2019) however, there is a need for comprehensive dementia education (Farina et al., 2020a). To date, no research has looked at dementia eduction through a pedagogical lens. This is essential in order to develop dementia education within the curriculum. Therefore, this paper aims to explore how pupils learn about dementia through a pre-planned educational session in the primary classroom. In so doing, it contributes to the possibility for improving our understanding of the pedagogical approaches required for effective dementia education.

### Developing an effective pedagogy of dementia education for primary-aged pupils

It has been debated that effective dementia education requires an affective, embodied approach to pedagogy to capture the complex and multifaceted experience of dementia (Brown et al., 2018; Surr et al., 2017). Recent developments in primary education have seen a shift from traditional methods of learning towards embodied approaches to learning, the engagement of the whole body in learning, drawing on and evoking sensorimotor responses (Engel et al., 2013). Engaging the body in learning experiences creates opportunities to engage the senses and bodily responses (Probyn, 2004), and opens the opportunities to create meaningful knowledge for the learner beyond the traditional quiet engagement in mental tasks (Macedonia, 2019). It sets a challenge for the teacher to find ways of ‘scaffolding’ or extending learning by considering carefully the intended knowledge or skills to be developed, whilst providing a rich and stimulating environment that allows learners to explore and experiment on their own terms with a variety of different resources––both human and non-human (Fenwick et al., 2011).

Embodied learning involves planning for educational experiences that encourage interaction between both human and nonhuman things as a relational endeavour (Dixon & Senior, 2011; Klykken, 2022; Mulcahy, 2012). Providing opportunities to interact with things in a variety of ways can enhance the learning experience and create new understandings of the subject matter, for pupil and teacher alike, as educators are encouraged to attend to the different effects of educational processes (Fenwick & Landri, 2012).

Therefore, adopting an embodied learning approach could be useful for dementia education for children because focusing on the ‘felt’ bodily relationships opens a range of possibilities as to how the learner interacts with the material and the teacher and could help to develop an empathic understanding of dementia. In doing so, it encourages us to attend to the process of learning, as well as outcomes.

### Dementia education in Scotland

There is opportunity, as well as a rationale, to support dementia education more specifically within Scotland’s education system, which does not prescribe specific contexts for learning. ‘A Curriculum for Excellence’ (Education Scotland, 2019) is a national 3–18 framework that enables teachers to create experiences for learning with flexibility, encouraging practitioners to select contexts that are learner-centred and differentiated according to their knowledge and skills needs. Educational professionals can co-create a responsive curriculum that offers personalisation and choice for learners, rather than following mandatory subject-based guidance.

The four dimensions of ‘A Curriculum for Excellence’ (Education Scotland, 2019; Scottish Executive, 2006) focus on supporting young people to become a successful learner, a confident individual, an effective contributor and a responsible citizen. Of these, the goal of students becoming a ‘responsible citizen’ is most apt for a dementia education, where teachers are asked to provide experiences and educational outcomes which *develop particular attributes (such as ‘respect for others’) and capabilities (such as ‘develop[ing] informed, ethical views of complex issues’ and ‘evaluat[ing]…scientific issues’ –* Education Scotland, 2019*, np).* Unlike other nations within the UK – where citizenship is a taught-subject – Scotland has instead encouraged an approach which allows teachers and educational practitioners to decide upon the context for teaching these capacities, such as through *other subjects, including Science and Health and Wellbeing.* Here, we can see the possibility for including a dementia education project which relates to developing responsible citizens, and both supports knowledge and understanding of dementia, whilst encouraging young people to build intergenerational understanding through an empathic approach.

### Understanding dementia: Class in a bag

The University of the West of Scotland has developed an award-winning dementia education resource Understanding Dementia: Class in a Bag. This resource was co-produced with student nurses and lecturing in our undergraduate nursing programme, the development and motivations behind the initiative have been published elsewhere (McGarry et al., 2021). Understanding Dementia: Class in a Bag was originally developed to support student nurses to go out to schools to deliver sessions on dementia to pupils as part of their own education. The bag has since been developed into a marketed product and is available to buy with a facilitators handbook which is the version used in this research. The learning objective of the Class in a Bag session is to support children to become dementia aware, socially responsible citizens. The underpinning pedagogy of the bag is Care Empathia (McGarry et al., 2021), an approach which aims to change how people think, feel and what they do about dementia. The contents of the bag are designed to connect with the three domains of learning, cognitive, affective and psychomotor (Bloom et al., 1956). The Bag contains all the materials to run three short activities with the class, alongsidea context-setting storybook called Molly’s Story, which tells of the relationship between Mrs Brown who is living with dementia, and her 10-year-old neighbour Molly. The three activities are (1) understanding the brain, (2) understanding the experience of dementia and (3) how to help. The activities are explained in detail below. The full Understanding Dementia: Class in a Bag session, including introduction and Molly’s story takes around 80 minutes to complete. The research reported here used ‘Understanding Dementia: Class in a Bag’ to deliver the dementia education session.

## Methods

The research adopted a qualitative approach using video methods and focus groups. The research took place in a school in the west of Scotland, purposively selected due to a pre-existing relationship with the research team and university. A Primary 6 class (total 33 pupils) was selected as the participating class. Ethical approval was granted by the School of Education and Social Science Ethics Committee at University of the West of Scotland. A parental information sheet and consent form was provided to the parents/carers of pupils in the class. In addition, the pupils in the class were informed about the research through a ‘My Research Story’ presentation delivered in class by the researcher 2 weeks before the data collection and a printed version for them to take home to discuss with parents/carers. ‘My Research Story’ explained to pupils the role of the researchers and outlined the protocol for research in child-friendly terms. It also showed pupils how to ‘opt out’ of the research, while still being involved in the lesson. Participant information sheets explained that the videos and still images from the videos would be used for the research, but that no images would be used in publications which identify any of the participants. Consent forms were returned by parents prior to the day of the lesson and pupils were asked to indicate their assent to participate by thumbs up/down (Menter et al., 2011). Twenty-three consent forms were received and those pupils participated in the research (m = 12, f = 11; age 10–11 years). They were separated into two groups (*n* = 11; *n* = 12). Those who did not provide parental consent/assent (*n* = 10) were grouped together to allow them to participate in the lesson without engaging in the research.

### Stage 1: Recording the learning session

The class teacher led the session using the facilitator’s manual. The teacher and two researchers (authors initials) acted as facilitators for the session, each leading one activity. The activities are designed to be child-led and exploratory, so the facilitators role was to provide prompts on using the materials (for example, which order to put on the goggles and gloves in the activity on the experience of dementia) or to encourage the pupils to think about the relevance of the activity (for example, what the materials in the how to help activity were used for). Three activities were set up in the classroom (understanding the brain, understanding the experience of dementia and how to help) and the pupils moved around each of the activities in their groups. Approximately 20 min was spent at each activity. The pupils were given two iPads per group to film their experiences during the session. They passed the iPads around, taking turns filming and using the materials in the bag. Participatory video enables a unique insight into the ‘invisible’ connectivity of children’s everyday lives with limited intrusion from adults: it allows children to focus on what is interesting to them, as well as providing more control and ownership for the children regarding their learning within the research project (Lomax et al., 2011). The pupils recorded their learning activities in real-time as they were participating in the session (Meager, 2017) which allowed pupils to provide focus on what they found interesting and the important parts of the lesson to them, which is of paramount importance in participatory, child-centred methods (cf. Clark & Moss, 2011).

The pupils recorded a total of 54 min and 36 s of film during the activities. These videos were recorded on four ipads and contained footage of both groups doing all three activities. The length of the video clips ranged from 11 s to 17 min and 8 s long. The videos pupils made were transcribed using a video log (Lawrence, 2020) which provided a scene-by-scene description of what was going on as well as a transcription of the dialogue captured on film. The video logs were created by [researcher] who was not present at the data collection session. Both researchers recorded field notes after the session. This included information about the classroom environment and how pupils were interacting with the materials and the iPads.

### Stage 2: Reflective focus groups

Two weeks after the session, the researchers returned to carry out focus groups with pupils to explore their thoughts, feelings and understanding of the session. There were two focus group discussions, each with 10 participants each, pupils were kept in the same groups as they had participated in during the session, three pupils were absent from school on the day of the focus group and one pupil decided not to participate in the discussion. The focus group schedule was developed using the video footage recorded by the students. The research team reviewed the footage and pragmatically selected sections of film which represented each of the tasks clearly and represented a variety of pupils across both groups. Pupils were invited to discuss what they were seeing and what they thought was happening. They were asked about likes and dislikes of the session, as well as how they would feel about meeting someone with dementia. Focus groups lasted between 40 and 50 min and were audio-recorded and transcribed. Pupils were asked to provide a synonym for use within the research to promote anonymity.

### Data analysis

#### Stage 1: Creating vignettes from the recording of the learning session

Data from video logs and fieldnotes were used to create analytic vignettes. Analytic vignettes are ‘compact sketches that can be used to introduce characters, foreshadow events and analysis to come, highlight particular findings, or summarise a particular theme or issue in analysis and interpretation’ (Ely et al., 1997: 70). Used commonly within arts-based and participatory forms of qualitative inquiry, analytic vignettes are used to explore key themes emerging from the data in ways that enable audiences to experience such themes vicariously (Jenkins et al., 2016).

In this study the vignettes were created using the video log data and field notes from the learning session. Data were grouped around each discrete activity within the CIAB session, these data were then categorised around key themes to create analytical vignettes which illustrate the experience of the learning sessions. initial transcription of the video logs and categorisation of was carried out by [researcher] who was not present at the session to minimise bias and risks of the facilitator perspective of the session influencing the analysis process. The research team then worked together to refine and draft the vignettes. The vignettes are not a description of a single point in the lesson, instead they are a composite of the experiences captured by the pupils to present a snapshot of the activity (Ely et al., 1997)*.* In creating these vignettes, we have replicated the process outlined by Jenkins et al (2021) and as far as possible edited together data extracts from our sources to provide a representation of the learning process, only changing tenses, or adding in contextual information for clarity.

#### Stage 2: Analysis of focus groups

Focus group data were analysed using content analysis (Elo & Kyngäs, 2008). Focus groups were transcribed verbatim and using the transcripts, data were first organised into categories related to each activity, then open coded and grouped into categories under headings such as brain health, learning about dementia, helping people, empathy and alternative uses for the materials. Categories were then refined and arranged into four higher categories presented below. Headings for these categories were created using the language of the participants to represent the voice of the pupils.

## Findings

### Stage 1: Vignettes from the learning session

**Figure 1.**
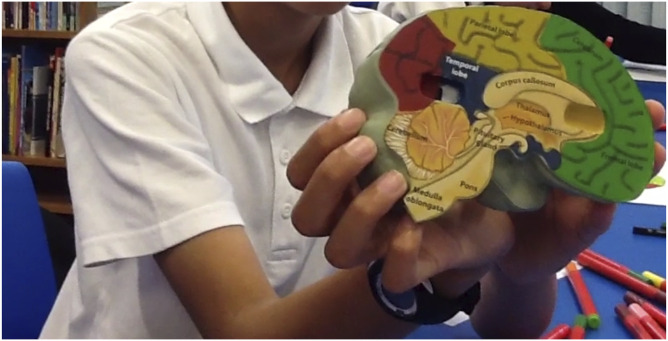
Still image of a pupil showing the model brain to the camera.

**Figure 2.**
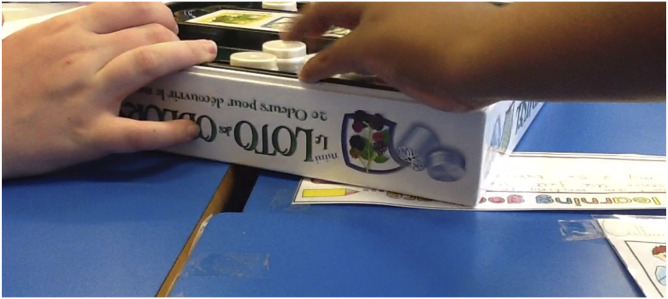
Still image of the pupils reaching for the box of smells.

**Figure 3.**
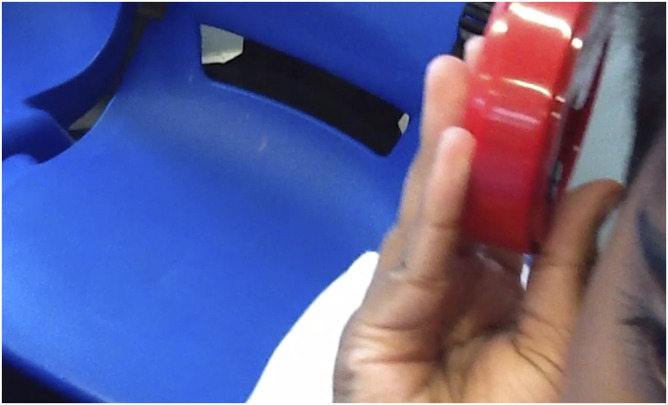
Still image of a pupil holding the talking product’s ‘Talking Tins’.

As described above, these vignettes were created to showcase the process of learning for the pupils in the class, demonstrating the experience of each activity (Figures 1, 2, and 3).

#### Activity 1: Understanding the brain

This session is designed to help pupils learn about the brain, how to keep the brain healthy and the physiology of the brain. Pupils are provided with a soft foam model brain in a box which has coloured sections to indicate the different areas of the brain.

### Vignette 1: ‘Your brain fell out’

The model brain is placed on the table and the pupils immediately grab for it, they are all keen to touch the model brain to see what it feels like. “I’ve not had it yet” one pupil complains. “The brain has divided” states another as the model comes apart to reveal a cross section.

“Is this our brain?” one child asks in amazement. One of the pupils picks up the model and squeezes it “It’s very squishy”. Another shouts “my brain’s gone weird!” holding up the model of the brain next to his own head. Soon after, he lets go of the model whilst simultaneously dropping his head sideways. “Oh no!” another child joins in, followed by laughter. “Your brain fell out!” adds another child.

The pupils have all had a turn of passing the brain about, each one touching and squeezing it in front of the camera. The camera then turns to the facilitator who asks “what do you wear when you go out on your bike?” and the pupils shout “a helmet” in response and “to protect our brains” when she asks why they wear a helmet. The follow up question of “is there anything else you could do to protect your brain?” is met by silence before the camera focuses again on the facilitator who is explaining that what keeps our body healthy also keeps our brain healthy. The camera then focuses on pupils in the group as they come up with answers like “exercise”, “drink water”, “don’t eat bad things” as answers to how we can keep our brains healthy.

#### Activity 2: Experiential learning activity

The second activity aims to provide pupils an understanding of what a person with dementia may experience. Pupils are provided with a set of goggles with stickers on the lens to obscure vision, gloves and an mp3 player with pre-recorded white noise. Pupils are instructed to put on the equipment, once they are ready, the facilitator presents a deck of cards and gives specific instructions to find a card from the deck.

### Vignette 2: “I didn’t understand!”

The camera focuses on a boy putting goggles, gloves and headphones, he’s smiling at the camera. The facilitator begins to introduce the activity and the camera turns to capture her saying “this task is to show how if your senses are impaired it can be difficult to do everyday tasks.”

She then hands the boy a pack of cards and asks him to find the 3 of clubs and the 6 of diamonds. He repeats “3 of clubs, 6 of diamonds”. He then asks “three of any clubs?” He has misunderstood the instructions. He proudly shouts “I’ve got it, I’ve got it!” but his friend leans across the table, holds up the six of diamonds and says “no, six of diamonds, not six diamonds.”

They laugh, as he says “right I’ve got it now” but seconds later he scans the cards on the table to look for the card and he is lost again as he can’t find it. After the boy has removed the goggles, gloves and headphones, and passed them on to another pupil, he can be heard saying to the facilitator “I didn't understand, I thought you meant like six of ANY diamonds...”.

#### Activity 3: Things that help

The third activity is a range of products which might help a person with dementia. There are two vignettes related to this activity, this first focussed on a box of scent pots that are designed to be used as a reminiscence activity. The second part is a range of low tech solutions which could help a person maintain independence within their home, for example a large button telephone and a talking tin lid.

### Vignette 3: ‘Everyone likes orange’

The camera is positioned on the table with the box of scents in view. Lots of hands come into shot, each one grabbing a pot out of the box and the pupils can be heard excitedly shouting “what’s this one?” “what’s this?”. The excitement is clear, and the camera continues to film hands moving excitedly back and forward as the pupils take the different pots, smell them and put them back.

The pupil who has control of the iPad continues to film and show close-ups of the pots he picks, “this one is hay” he says. In the background, there is lots of chatter about the different smells and stories about what they mean to them.

Individual preferences start to emerge, one pupil thinks the chocolate is horrible, while another thinks everyone likes orange only to find someone else thinks it’s disgusting.

The pupils are starting to relate smells to their own lives and telling stories, one scent leads to a conversation about seasoning used for Polish dumplings. Another tells a funny story about his Mum setting a steak on fire using a sprig of thyme when he smells the thyme pot. The camera then focuses on the facilitator who talks about what these smells might mean to a person with dementia, giving an example that they might remember the smell of furniture polish from when they were a child. The pupils seem to understand that smells can trigger memories. “It’ll help their brain”, they say in response to a question about why this might be important for people with dementia.

### Vignette 4: ‘This is genius’

It’s time to look at the technology in the bag. “Here’s the telephone, oh, we need to put it together” the facilitator says as she takes it from the bag and passes it to a girl to assemble. The facilitator and pupils have a joke about how difficult the phone is to put together––“this is part of the test” the facilitator jokes, but the pupils are repeatedly saying “this is a hassle”. The facilitator starts to ask questions about why someone with dementia would use a phone like this and the pupils are able to answer the questions easily. They understand that the big buttons will help someone with dementia to be able to see them better, and that the pictures will help them phone someone without having to remember the number. The pupils start playing with the phone, filming each other as they phone different people. One child is trying to phone his Mum, another phones herself.

Next, the facilitator introduces the hot drink indicator made by the RNIB, and a pupil reads out the back of the packaging to the group: “Make a hot drink with more confidence by placing it on the side of your cup when the cup is nearly full it will let me know with beep sounds…”. At first the pupils can’t quite understand what this is for, but they quickly realise it’s to stop someone pouring too much into a cup and they lose interest and turn the camera back to the phone.

The Talking Products’s ‘Talking Tins’ is introduced as the last item and attention turns away from the phone again. The facilitator is filmed showing the pupils how it works by pressing the button and recording your voice; the pupils come up with a range of ideas of how this could be used by a person with dementia. “This is genius” one girl shouts, another records a message on it saying ‘mum it’s night-time you can’t go out’. They are really excited about the potential this product has for helping a person with dementia, even though the intended use is to help recognise tins of food. They all want a shot of it now. Just as the pupils are passing around the tin lid, the facilitator announces the session is about to end, so the pupils all wave goodbye and shout “bye camera”.

### Stage 2: Focus group findings

#### Category 1: It’s more than forgetting things

In the focus groups, pupils reflected on what they had learned from the session. Many pupils spoke about how the activities helped them to understand what dementia is and that it was more than just forgetting things. The brain activity appeared to influence this by allowing the pupils to see different areas of the brain and begin to make an association between the brain and dementia.

I liked when we were having the model of the brain because you can understand about what dementia is it, it was quite interesting to look at what parts of the brain get affected when people have dementia. (Sophia, FG1)

The other activities, particularly the goggles and the smells introduced the sensory element of dementia to the pupils and helped them to realise dementia affects more than just memory.

*…because I only thought that they forget things but then it effects their senses and everything*. (Jenny, FG1)

#### Category 2: I feel a bit bad for people with dementia

The goggles activity seemed to be the most popular with the pupils and generated a lot of discussion in the focus groups. The pupils identified that the activity helped them to understand what a person with dementia might experience and were able to use the activity to imagine how a person with dementia might feel.

They might see it differently as us, we see it quite normally and they see it quite hard because they don't know, they don't know what they’re doing anymore because of their dementias (Charlie, FG2)

Watching back the footage of the experiential session (as described in vignette 2) allowed the pupils to discuss what they felt during the session.

It was quite hard and it was actually with the gloves and just a wee bit of noise and the goggles, it was quite easy but then when the noise got really, really loud, I couldn't actually do it and I just threw the headphones off enraged. (Jordy, FG2)

This allowed the pupils to explore how the activities, while seemingly simple for them, caused feelings of frustration and confusion.

I think he didn't like it because you usually do it very good and then he cannot so he thinks that he’s bad at it. (Bob, FG1)

This in turn allowed the pupils to relate this experience to empathise with people who are living with dementia.

I felt quite empathetic because I was like imagining what it would be like to like, live with a person with dementia. (Bobby, FG1)

#### Category 3: I can help my Grandad.

Throughout the focus group discussions, the pupils related their learning to how they could help people with dementia. Some of the pupils had relatives with dementia, and they felt more confident in how they could approach their relatives:

I learned a lot of new things and how I can help my Grandad if I was ever around him. (Geraldine FG 1)

I told my Mum and my Dad and he said it was really good so you can understand more about what’s going on with my granddad and everything. (Billy, FG2)

Using the telephone and the other low-tech devices also helped the pupils to discuss their learning about how devices like this could help a person with dementia. The pupils could all see the relevance of the telephone and could see exactly how this would be useful:

You see the phone one with the images, yeah. I think it will help the people with dementia because if they forget a number they can just press the button with the picture. (Panda, FG2)

The numbers were really big because with the goggles that we wore in the third group that we were in, the eyesight was quite bad and it was quite hard to see so with the big numbers it was a lot easier to see for people with dementia. (Bobby, FG1)

However, as shown in vignette 4, the pupils began to use creative ways of applying their knowledge about dementia to come up with other ways of using the materials in the bag. This discussion carried on in the focus groups to the extent that the pupils discussed the red Talking Tin as a device that would help discourage people with dementia leaving the house at night.

*Albert: My favourite bit was the voice-recorder you could record your voice and then say someone with dementia always went out at night time thinking it was day so you could put the message on that recorder said "You can't go outside, Mum, it's night-time", so it's kind of to remind them…..I kind of disliked how instead of- I think it should have when it detects movement, instead of you having to push a button, because what if the person forgets to push the button?*

While this isn’t the purpose of the tin lid, the discussion in the focus groups shows how the pupils were keen to think about how to make a difference in the lives of people with dementia and use their learning to come up with innovative ideas. Similarly, the use of the box of scents was an activity that the pupils enjoyed but began to apply a different meaning to than the intended use as a reminiscence activity. They then went on to discuss alternative uses to help the person in everyday life.

I’d like, if they’re going to the shop and they’re like 'ooh, I don't know what to get' they like smell it and like 'that smells like that, so I could get that' (Geraldine FG1)

##### Category 4: How I can help myself

Throughout the activities, the pupils developed an understanding of brain health and how to reduce the risk of developing dementia in later life. One pupil highlighted that learning about dementia has increased their worry about developing dementia in later life, particularly as there is no cure for dementia:

*Maybe it's good a wee bit… but if it scares some person because they get dementia sometimes of people but they don't get better but if people are scared to get dementia afterwards.* (Bob, FG1)

However, the pupils discussed how by understanding more about dementia, they could do things to reduce their chance of getting dementia by drinking water, exercising, keeping fit and eating well and they pupils felt confident to share their new knowledge with each other and their family members to help them keep well.

You could say to them that they’re like, even if they didn't have dementia. If there was an old person, just tell them like maybe just say to them you are getting old and maybe like... stop smoking or like not drink alcohol and that. (Sophia, FG1)

## Discussion

This paper has provided important insights into how pupils learn about dementia through a pre-planned educational session in the primary classroom using the ‘Understanding Dementia: Class in a Bag’ resource. The findings have relevance to how we develop dementia education in the future and highlights the positive impacts of learning about dementia in primary school. The findings show that dementia education not only empowers children to talk about dementia, but it increases confidence in how they might interact with or support a person living with dementia in the future, as well as providing vital knowledge about how to reduce their chance of developing dementia in later life.

The vignettes demonstrate the learning experiences the pupils had whilst participating in the Understanding Dementia: Class in a Bag session. By providing pupils with iPads to record their own experiences we are provided with an ‘in the moment’ account of the interactions between the pupils and the materials in the Bag which enhances our understanding of the process of learning. The focus groups provided the pupils with an opportunity to discuss their learning from the session and helps us to understand the outcomes of the learning. By combining the two sources of data, this paper presents a unique insight into what works in dementia education allowing us to make recommendations for future educational initiatives.

Because dementia is a complex condition, a full understanding needs to consider the bio-psycho-social and environmental influences on the condition (Brown et al., 2018; Hvalič-Touzery et al., 2018; Surr et al., 2017). This includes developing an understanding of the underlying biological changes in the brain as well as the social aspect of dementia, such as fostering an understanding of the lived experience of dementia and how to help a person with dementia (Baker et al., 2019).

The vignettes show how learning about dementia is developed over the activities. Beginning with a biological understanding of the brain, pupils then develop an understanding of how the symptoms of dementia can impact an individual and finally, the social aspects of dementia and understanding ‘how to help’. Adopting a biopsychosocial approach to dementia is useful for understanding the human experience of living with dementia and helping to influence person-centred support and care (Keady et al., 2013). The focus group findings show that pupils had a deeper, empathetic understanding of dementia following the education session. While there are limitations to the biopsychosocial model of dementia, this study has shown, that using the biopsychosocial model for dementia education is useful for introducing dementia to primary school-aged pupils. Future educational initiatives and research could build on these findings and incorporate further cultural and environmental elements to the pedagogical approaches, particularly drawing on the growing interest in intergenerational research involving people living with dementia in educational initiatives (Gerritzen et al., 2020).

Moreover, the materials from the Bag play an important role in engaging the whole body in the learning experience, supporting pedagogical approaches which encourage embodied forms of learning (Dixon & Senior, 2011; Macedonia, 2019). Often interacting alongside a peer, pupils were interested in more than their aesthetic complexities and gained enjoyment from how things moved, sounded, felt, and smelled: they experienced the ‘unexpected’ as well as the familiar, developing new understandings of how previously known materials might be experienced differently by someone with dementia. In addition, through an embodied approach to learning, this study aligns with others that advocate a hands-on, experiential approach to unfamiliar (eg Satterthwait, 2010) or challenging issues (Henderson & Dombrowski, 2018; Trofanenko, 2011). Our study has shown how even a short session on learning about dementia can be impactful in the sense that some pupils reported changes in their approach to the condition, whether this be increasing worry for getting this in the future, considering how to improve their own and others’ lifestyles to lessen its impact and thinking about how to support a person living with dementia who is known to them.

The interactions between the pupils, facilitators and materials from the bag are at the heart of the learning in this study, allowing the pupils to play, enjoy and explore in their own ways. By focusing on the social and material relations that comprise the things of the classroom, we can begin to understand how learning is an embodied process, where knowledge and understanding emerge as it is experienced by the pupil and challenging our conceptions of how we define ‘pupils’ and ‘teachers’ (Ellsworth, 2005; Roehl, 2012). Moreover, the ‘things’ used in learning environments, including natural and non-natural resources, are important to attend to in order to understand how the pupils interact with the materials, and how the relationship between the human and non-human elements creates meaning (Fenwick & Landri, 2012; McGregor, 2004).

The strength of this study is that we have attempted to view the learning from the perspectives of the pupils. Providing them with iPads to film the session allowed the pupils to record what they found interesting and important. The initial excitement of using the ipads in this way was clear, however from the outset the research team created clear guidelines for using the iPads (i.e., to take turns and only to use the camera/video function during the session) and sharing the filming between the group members. However once the activities started the pupils were keen to share the filming to ensure they all got to try the activities. The iPads became part of the activity for the pupils with one group using the iPad to create a news style report of the activity. Although at times the technology may have distracted from the task, the video data provides depth to help us understand and interpret the process of learning while still giving the pupils a feeling of control over their environment (i.e., they chose how and what to film).

Yet, what this paper has not concluded is the contribution of the spaces of learning to pupils’ affective and embodied experiences of learning about dementia. Future research studies might explore how the layout of a classroom, or the time of day, affect pupils’ engagement with the materials. And whilst this paper has outlined a largely positive experience of pupils learning about a challenging and often unfamiliar subject, Bob’s worried response to the lesson is a caution to other educators to ensure that they take full cognisance of aspects that might evoke an emotional response (see also Probyn, 2004, 29–30). Although Bob expressed a worry about developing dementia, they were not distressed and engaged a discussion on dementia prevention and brain health during the focus group. Following the focus group, further resources which could provide support and information were provided to the class teacher for dissemintation.

Similarly, this study does not explore the long-term impact of the session or how dementia education can be integrated into the primary curriculum in an engaging way but instead highlights that dementia can be a topic that engages pupils, promotes wellbeing and that pupils enjoy in a way that aligns with the Scottish Curriculum Framework (CfE). Whilst some commentators have praised the Curriculum for Excellence’s open-ended approach for its responsiveness to local circumstances and learner-centredness, others have argued that this means that important contexts for learning – like Human Rights Education – can be legitimately side-lined (Daniels, 2018), and that the types of citizens that the capacities encourage are individualistic, and lacking an awareness of pluralism, where “it is important, therefore, to be precise about the nature of the activity and the domain in which the activity is exercised” (Biesta, 2008, p. 46). Therefore, this study provides valuable insights into how dementia education can operate, especially where there is limited knowledge on how children understand and learn about dementia. However, the CIAB session used in this study was pre-determined in terms of the content covered in the activities, although this broadly aligns with previous research exploring what children need to know about dementia (Baker et al., 2018) more research is required to fully explore the materials used in dementia education and wider curriculum links. For instance, the increased attention on dementia prevention and promoting brain health in public health strategies (see Brain Health Scotland, 2022 for example) may warrant further research in terms of how this is communicated in schools.

Further to this, this study highlights that dementia education has a role to play in responding to United Nations Sustainable Development Goals to promote global citizenship education (SDG4), and the need for initiatives to reduce the risk of preventable diseases (SDG3) (UN, 2015). Firstly, by helping children to develop an understanding of dementia so that they are more confident in being around and helping people with dementia both now and in the future. This element of global citizenship, where children are empowered to make changes in society as they grow, could help to improve the lives of people living with dementia and reduce stigma associated with the disease (Farina et al., 2020a). Secondly, dementia education has an important role in reducing the risk of dementia in later life (WHO, 2021).

## Conclusion

This paper has shown the possibility of learning about dementia in a primary education classroom, where a ‘hands-on’, embodied pedagogical approach can help pupils develop an affective, empathetic response to those who are experiencing the condition. The study has implications for how dementia education providers should develop and promote lessons about dementia to primary teachers, where even playful activities require sensitive contextualisation and a consideration of emotional safety, particularly given that some pupils may become anxious about their own or family members’ development of the condition. Whilst we argue that more research is needed to evaluate teachers’ experiences of dementia education, this study has also shown that dementia education has a place in the primary school curriculum, where Scotland’s *Curriculum for Excellence* enables child-centred lessons relating to citizenship, health and wellbeing, and science education. At the same time, where teachers – such as those in Scotland - have autonomy to decide which contexts to develop to meet educational outcomes, challenging or unfamiliar topics such as dementia education may be sidelined, particularly in an increasingly cluttered curriculum with competing demands. This study has importance, therefore, in demonstrating to an international audience that dementia education is viable in the school system. This is especially important where it is not already operating, where its prevalence is unknown, or where its appearance might be restricted by more rigid curriculum outcomes.
